# NF-κB-Mediated CCL20 Reigns Dominantly in CXCR2-Driven Ovarian Cancer Progression

**DOI:** 10.1371/journal.pone.0164189

**Published:** 2016-10-10

**Authors:** Rosa Mistica C. Ignacio, Syeda M. Kabir, Eun-Sook Lee, Samuel E. Adunyah, Deok-Soo Son

**Affiliations:** 1 Department of Biochemistry and Cancer Biology, Meharry Medical College, Nashville, Tennessee, United States of America; 2 Department of Pharmaceutical Sciences, College of Pharmacy, Florida A&M University, Tallahassee, Florida, United States of America; Seoul National University College of Pharmacy, REPUBLIC OF KOREA

## Abstract

Ovarian cancer is an inflammation-associated malignancy with a high mortality rate. CXCR2 expressing ovarian cancers are aggressive with poorer outcomes. We previously demonstrated that CXCR2-driven ovarian cancer progression potentiated NF-κB activation through EGFR-transactivated Akt. Here, we identified the chemokine signature involved in CXCR2-driven ovarian cancer progression using a mouse peritoneal xenograft model for ovarian cancer spreading with CXCR2-negative (SKA) and positive (SKCXCR2) cells generated previously from parental SKOV-3 cells. Compared to SKA bearing mice, SKCXCR2 bearing mice had the following characteristics: 1) shorter survival time, 2) greater tumor spreading in the peritoneal cavity and 3) higher tumor weight in the omentum and pelvic site. Particularly, SKCXCR2-derived tumor tissues induced higher activation of the NF-κB signaling pathway, while having no change in EGFR-activated signaling such as Raf, MEK, Akt, mTOR and Erk compared to SKA-derived tumors. Chemokine PCR array revealed that CCL20 mRNA levels were significantly increased in SKCXCR2-derived tumor tissues. The CCL20 promoter activity was regulated by NF-κB dependent pathways. Interestingly, all three κB-like sites in the CCL20 promoter were involved in regulating CCL20 and the proximal region between -92 and -83 was the most critical κB-like site. In addition, SKCXCR2-derived tumor tissues maintained high CCL20 mRNA expression and induced greater CCL24 and CXCR4 compared to SKCXCR2 cells, indicating the shift of chemokine network during the peritoneal spreading of tumor cells via interaction with other cell types in tumor microenvironment. Furthermore, we compared expression profiling array between human ovarian cancer cell lines and tumor tissues based on GEO datasets. The expression profiles in comparison with cell lines revealed that dominant chemokines expressed in ovarian tumor tissues are likely shifted from CXCL1-3 and 8 to CCL20. Taken together, the progression of ovarian cancer in the peritoneal cavity involves NF-κB-mediated CCL20 as a main chemokine network, which is potentiated by CXCR2 expression.

## Introduction

Ovarian cancer is the fifth leading cause of cancer death among women. Because it is typically asymptomatic, ovarian cancer has been usually detected at late stage when tumors have spread far beyond the ovaries [1]. Ovarian cancer is recognized as an inflammation-associated cancer [2] and cancer cells express high levels of tumor necrosis factor-α (TNF) as a potential regulator of the proinflammatory tumor microenvironment [2–4]. Our previous studies demonstrated that TNF activated the nuclear factor-κB (NF-κB) signaling to induce proinflammatory chemokines such as CCL20, CXCL1-3 and CXCL8 in ovarian cancer cells [5–7]. Chemokines contribute to cancer progression and metastasis as critical mediators in the tumor microenvironment [8, 9]. Particularly, ovarian cancer cell lines express high levels of proinflammatory chemokines CXCL1-3 and CXCL8 [6, 7] as specific ligands for the chemokine receptor CXCR2 [10]. The CXCR2 was frequently expressed in tumors obtained from patients with ovarian cancer, prompting ovarian cancer progression [11]. Other cancer types also indicate that CXCR2 is closely associated with cancer progression. CXCR2 knockout mice significantly reduced tumor burden in some cancer models such as prostate cancer [12], lung cancer [13] and renal cancer [14]. Inhibition of CXCR2 suppressed inflammation-driven tumorigenesis in skin and intestine [15]. The absence of CXCR2 prevented colon cancer cell growth [16] and CXCL1, a CXCR2 ligand, was inversely associated with recurrence-free survival in colorectal cancer patients [17]. These facts support a critical role of CXCR2 in ovarian cancer progression.

CXCR2-mediated signaling is known to exert multiple pathways such as anti-apoptosis, EGFR activation and angiogenesis [11–16]. In our recent study, potentiating NF-κB activation through EGFR-transactivated Akt augmented proinflammatory chemokines CXCL1/2, contributing to CXCR2-driven ovarian cancer progression [18]. Moreover, as described in detail previously [18], the molecular mechanism involved in the cancer progression indicates the significant role of NF-κB signaling, a main proinflammatory pathway, on the potential contribution of CXCR2 in ovarian cancer progression. Based on parental SKOV-3 ovarian cancer cell line, we generated stable CXCR2 transfected cells (SKCXCR2) as well as control cells transfected with empty vector (SKA) as described previously [18]. Here, we employed the mouse peritoneal spreading model for ovarian cancer and identified a main signaling pathway and chemokine network involved in CXCR2-driven ovarian cancer progression using tumor tissues of the omentum, a main metastasis site for ovarian cancer. Furthermore, based on Gene Expression Omnibus (GEO) datasets, the present study demonstrated that the chemokine network could be altered during the progression of ovarian cancer such as peritoneal tumor dissemination and massive ascites via interaction with other cell types including immune cells and stromal fibroblasts in the tumor microenvironment.

## Materials and Methods

### Reagents

Recombinant human TNF was obtained from R&D Systems (Minneapolis, MN). Antibodies were purchased as follows: CXCR2 (E-2, sc-7304) and β-actin were from Santa Cruz Biotechnology (Santa Cruz, CA) and IκB, EGFR, Erk1/2, Akt, Raf, MEK, mTOR and their phosphorylated forms such as pIκB (Ser32/36), pEGFR (Tyr1173), pErk1/2 (Thr202/Tyr204), pAkt (Ser473), pB-Raf (Ser445), pc-Raf (Ser338), pMEK (Ser217/221) and pmTOR (Ser2448) were from Cell Signaling Technology (Beverly, MA). Lipofectamine 2000 and all liquid culture media were acquired from Invitrogen (Grand Island, NY). A customized PCR array for the chemokine network, primers for CCL20 and CXCR4, and a SYBR^®^ Green Master Mix came from SABiosciences in Qiagen (Frederick, MD). Chemiluminescent detection kits were from Santa Cruz Biotechnology (Santa Cruz, CA). Antisense and sense oligonucleotides were obtained from Eurofins MWG Operon (Huntsville, AL). Finally, the Luciferase Reporter Assay System was obtained from Promega (Madison, WI).

### Generation of stable CXCR2 expressing SKOV-3 cell line and culture

The human SKOV-3 ovarian cancer cell line was purchased from the American Type Culture Collection (Manassas, VA). For this study, SKOV-3 cells were used to easily form ascites fluid as a main characteristic for metastatic ovarian cancer [19, 20]. CXCR2 expressing cell lines were generated by stably transfecting CXCR2 or empty vectors into parental SKOV-3 ovarian cancer cells and selecting G418-resistant clones as described previously [18]. The CXCR2 positive cell line was termed SKCXCR2, and the CXCR2 negative control cell line, SKA. Ovarian cancer cells (~5 X 10^4^ cells/ml) were cultured in RPMI medium containing penicillin/streptomycin and 10% FBS at 37°C in a water-saturated atmosphere of 95% air and 5% CO_2_. Treatments with reagents are described in detail in the Results.

### Mouse peritoneal xenograft model

Mouse peritoneal xenograft model was performed under institutional guidelines approved by the Institutional Animal Care and Use Committee at Meharry Medical College and adhere to the ARRIVE (Animal Research: Reporting *In Vivo* Experiments) guidelines for reporting animal research [21]. Immune-compromised female SHC^™^ nude mice (6 to 8 week-old, Charles River Laboratories, Wilmington, MA) were used for a peritoneal xenograft model. The mice were maintained in a specific pathogen free animal housing facility at 22°C±2°C and 40%–60% humidity under a 12:12 light: dark cycle. The mice were housed in groups of four per cage and allowed a week acclimation period before randomly assigned to the following groups: SKA (*n* = 8) and SKCXCR2 (*n* = 8). SKA and SKCXCR2 cells (each 3 X 10^6^ cells/mouse) were intraperitoneally injected into the peritoneal cavity of mice, respectively. Mice were monitored 3 times weekly to assess animal health such as hunched posture, lethargy and inactivity, impaired ambulation, shallow or labored breathing, hair coat condition and change in the body weight. In particular, mice showing clinical signs of ascites fluid production with constant increase of body weight and changes in appearance and activity were observed daily. When 20% increase in the body weight, extensive ascites accumulation or sluggish activity were observed, animals were terminated for humane reasons (S1 Fig). The outcome of mortality might be anticipated. There was some difficulty in judging both tumor burden and agility without extensive accumulation of ascites. In that case the peritoneal tumor burden could be observed only when dead mice were necropsied. At the end point, the mice were anesthetized with 2% isoflurane O_2_ mixture followed by cervical dislocation and the tumor spots in the abdomen were inspected in the peritoneal wall, liver, diaphragm, omentum and pelvic sites. Particularly, solid tumors from the diaphragm, omentum and pelvic sites were weighed for spreading index and prepared for RNA and protein studies as well as histological examination by fixation in neutral buffered formalin. The dead animals were necropsied and the extend degree of peritoneal spreading was observed. The survival time of the mice were compared between SKA and SKCXCR2 bearing mice.

### Western blot

Whole-cell lysates were prepared, fractionated on SDS-polyacrylamide gels and transferred to nitrocellulose membranes according to established procedures [18]. The following primary antibodies were used: EGFR, Raf, MEK, Akt, mTOR, Erk, IκB and their phosphorylated forms (Cell Signaling Technology, Beverly, MA). The protein bands were visualized by chemiluminescence detection kits. -Actin (Santa Cruz Biotechnology, Santa Cruz, CA) was detected as an internal loading control of cell lysates.

### PCR array and qRT-PCR

After isolating total RNA from tumor samples and eliminating genomic DNA, the RT reaction was performed at 42°C for 15 min followed by 94°C for 5 min. A real-time PCR reaction for chemokines was performed according to manufacturer’s instructions using a Bio-Rad CFX96 (Hercules, CA) and the following two-step cycling program: 1 cycle at 95°C for 10 min, and 40 cycles at 95°C for 15 sec and at 60°C for 1 min. Data analysis was performed based on a Web-Based PCR Array Data Analysis (http://pcrdataanalysis.sabiosciences.com/pcr/arrayanalysis.php) provided by SABiosciences in Qiagen (Frederick, MD).

### Construction of the CCL20 promoter and its κB-like site mutants

Based on CCL20 (-376/+20) promoter generated previously [6], we found three κB-like sites in CCL20 promoter and generated mutant constructs by mutating each κB-like site. Primers for mutation of κB-like site (italicized letters) were designed as follows: 5’-AGA ATT TAA CA***A* GAT TCT C*T*C** CTT CTC AAC-3’ for -219/-210 κB-like site; 5’-GAT CAA TG***A* GGA AAA C*T*C** CAT GTG GCA ACA-3’ for -92/-83 κB-like site; and 5’-ATA AAT A***A*G GCC ATC *T*C**A GGC TGC TGT CAG-3’ for -33/-24 κB-like site. The mutation of κB-like sites was performed by PCR-based mutagenesis using a site-directed mutagenesis kit according to manufacturer's instructions (Stratagene, La Jolla, CA). The mutant constructs of the CCL20 promoter were confirmed by DNA sequencing analysis.

### Transient transfection and luciferase assay

CCL20 promoter activity was performed with generated CCL20-376LUC vector and its mutants as previously described [6]. Ovarian cancer cells at approximately 50% confluency in 24-well plates were washed once with fresh media without additives and then transiently transfected with target vectors for 24 h at 37°C using Lipofectamine solution. Transfected cells were treated as outlined in Results and incubated for 6 h. After rinsing cells with cold PBS and adding lysis buffer (Promega, Madison, WI), cell lysates were used for determination of luciferase activity using a microplate luminometer. Luciferase activity, expressed as relative light units, was normalized to measured protein levels.

### Data analysis from GEO dataset

Data analysis was performed using microarray data sets deposited in the NCBI Gene Expression Omnibus (GEO, http://www.ncbi.nlm.nih.gov/geo/) database under accession number GSE34615 and GSE40595. Raw microarray data for chemokine network were RNA expression levels in 41 human ovarian cancer cell lines for GSE34615 and laser capture micro dissected ovarian tissue obtained from 8 microdissected normal ovarian stroma (NOS), 31 ovarian cancer stroma (OCS), 6 ovarian surface epithelium (OSE) and 32 epithelial ovarian cancer (EOC) for GSE40595 [22]. In analysis of cell lines, we used 29 ovarian cancer cell lines confirmed after removing cross-contaminated or misidentified cell lines among 41 cell lines [23]. We employed Gitools 2.2.3 (http://www.gitools.org) based on Oracle Java 7, an open-source tool to perform Genomic Data Analysis and Visualization as interactive heat-maps [24].

### Statistics

Data were analyzed by the paired Student’s *t*-test and one-way analysis of variance (ANOVA) as appropriate. If statistical significance (p≤0.05) was determined by ANOVA, the data were further analyzed by Tukey’s pairwise comparisons to detect specific differences between treatments.

## Results

### CXCR2-positive ovarian cancer cells spread extensively in the peritoneal cavity

**Fig 1:**
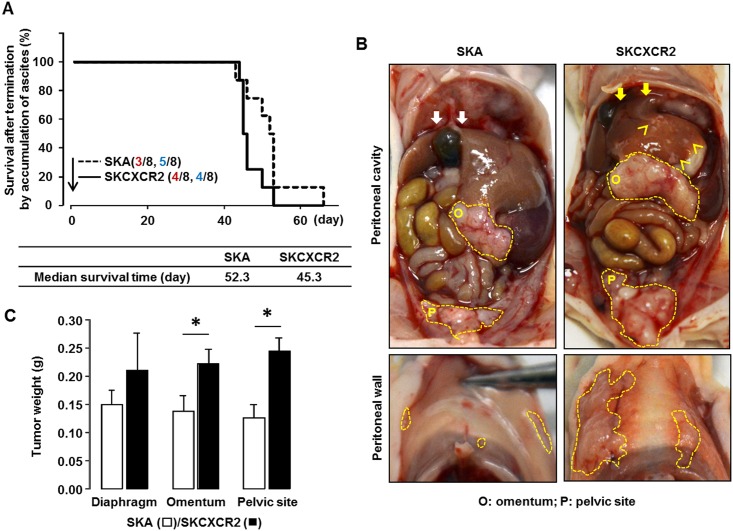
SKCXCR2 cells spread more extensively in the peritoneal cavity of mice as compared to SKA cells. (A) Survival time in mice bearing SKA vs. SKCXCR2 cells. Red numbers indicate the number of dead animals and blue numbers, the number of animals terminated by the accumulation of ascites. (B) The peritoneal spreading tumor burden in mice bearing SKA vs. SKCXCR2 cells. Closed dot lines indicate tumor tissues, white and yellow arrows show detachment and attachment between the diaphragm and the liver, respectively, and carets indicate tumor spots on the liver. (C) The weight of tumor tissues in the diaphragm, omentum and pelvic site obtained from mice bearing SKA vs. SKCXCR2 cells. Sample size was 5 SKA-bearing mice and 4 SKCXCR2-bearing mice terminated by accumulation of ascites and all data are shown as mean ± SE. * indicates a significant increase (p≤0.05) using Student’s-*t* test.

After intraperitoneally implanting CXCR2-negative (SKA) and positive (SKCXCR2) cells, we compared the extent degree of tumor burden. Throughout the experiment of peritoneal spreading model, we observed 3 dead and 5 terminated by the accumulation of ascites in SKA-bearing mice and 4 dead and 4 terminated in SKCXCR2-bearing mice. The cause of death was thought to be due to tumor burden based on necropsy. Mice bearing SKCXCR2 cells had shorter survival time (median 45.3 days) as compared to mice bearing SKA cells (median 52.3 days) (Fig 1A). SKCXCR2-bearing mice had more extensive spreading in the peritoneal cavity as demonstrated in the diaphragm, omentum, pelvis area and peritoneal wall (Fig 1B). Furthermore, SKCXCR2-bearing mice showed attachment between the diaphragm and the liver whereas SKA-bearing mice had detachment (Fig 1B). Tumor spots on the liver indicated aggressive spreading in SKCXCR2-bearing mice (Fig 1B). The weight of tumor tissues in the omentum and pelvic site was significantly higher in SKCXCR2-bearing mice (Fig 1C). On the other hand, there was no significant difference in the weight of tumor tissues in the diaphragm (Fig 1C).

### NF-κB signaling is significantly activated in the omental tumor tissues from SKCXCR2-bearing mice but no change in EGFR-downstream signaling

**Fig 2:**
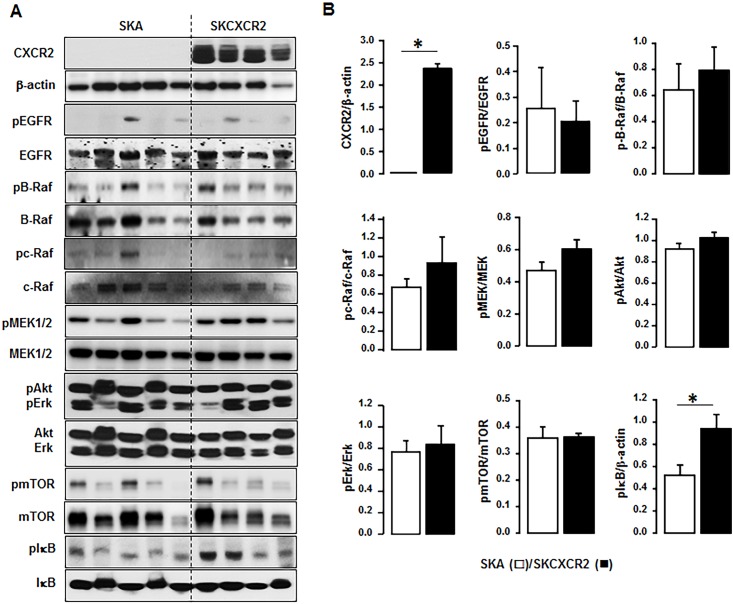
NF-κB signaling is significantly activated in the omental tumor tissues from SKCXCR2-bearing mice. (A) Comparison of signaling pathways in the omental tumor tissues from mice bearing SKA vs. SKCXCR2 cells. The pEGFR (tyr1068), pB-Raf, pc-Raf, pMEK, pAkt, pErk pmTOR and pIκB indicate phosphorylated EGFR, Raf, MEK, Akt, Erk, mTOR and IκB, respectively. β-Actin was used as a loading control. (B) Densitometric comparison of EGFR, Raf, MEK, Akt, Erk, mTOR and IκB activations in the omental tumor tissues from mice bearing SKA vs. SKCXCR2 cells. All data are shown as means ± SE. * indicates a significant increase (p≤0.05) by Student’s-*t* test.

As described in detail previously [18], CXCR2 positive ovarian cancer cells exert highly activated EGFR-downstream signaling such as Akt and Erk and NF-κB, compared to CXCR2 negative cells. Therefore, we compared these signaling pathways in proteins isolated from the tumor tissues of the omentum, a main metastasis site for ovarian cancer in the peritoneal cavity [25]. CXCR2 expression was confirmed in samples from mice bearing SKCXCR2 cells (Fig 2A). There was no difference in EGFR-activated signaling such as Raf, MEK, mTOR, Akt and Erk between SKA- and SKCXCR2-bearing mice (Fig 2A and 2B). Interestingly, the omental tumor tissues from SKCXCR2-bearing mice expressed higher IκB activation compared to those from SKA-bearing mice (Fig 2A and 2B).

### Chemokine network analysis reveals a specific increase of CCL20 in the omental tumor tissues from SKCXCR2-bearing mice

**Fig 3:**
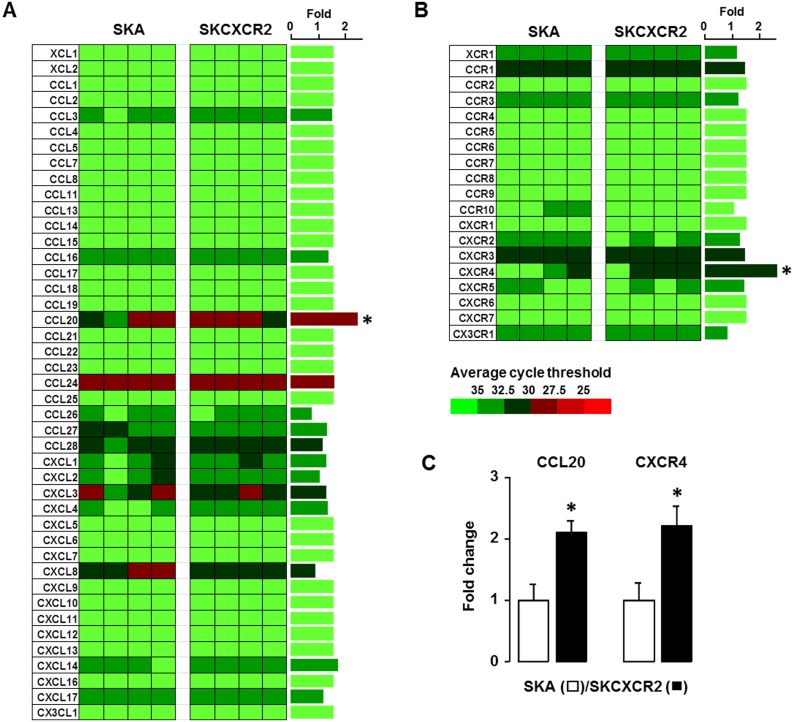
CCL20 is significantly increased in the omental tumor tissues from SKCXCR2-bearing mice. (A) Chemokine and (B) chemokine receptor network in the omental tumor tissues from mice bearing SKA vs. SKCXCR2 cells. After isolating total RNA and choosing the qualified RNAs for array, a human chemokine PCR array was performed. Different colors indicate the average cycle threshold with expressions that ranged from >35 to <25. Expression levels of chemokines were defined as absent (>35), low (30–35) and high (<30) on average threshold cycles. Chemokines with a >2-fold increase and average cycle threshold <30 are recognized as induced chemokines, and in this case represents CCL20 (*). (C) Confirmation of CCL20 and CXCR4 mRNA expression in the omental tumor tissues using qRT-PCR. Fold changes were calculated as a relative value after setting the average of SKA tumor tissues as a control group (1.0). All data value are presented as mean ± SE. * indicates a significant increase (p≤0.05) by Student’s-*t* test.

We further identified chemokine signatures by which NF-κB activation affects in the omental tumor tissues from mice bearing SKA vs. SKCXCR2 cells. We isolated total RNA (A260/A280 = >2.0) from tumor tissues of the omentum between SKA- and SKCXCR2-bearing mice and performed chemokine PCR array. The omental tumor tissues from SKCXCR2-bearing mice expressed higher mRNA levels of CCL20 in chemokines and CXCR4 in chemokine receptors compared to those from SKA-bearing mice (Fig 3A and 3B). Furthermore, we confirmed these results by qRT-PCR analysis (Fig 3C).

### The CCL20 promoter has three κB-like sites and the proximal region between -92 and -83 is most critical in regulating CCL20

**Fig 4:**
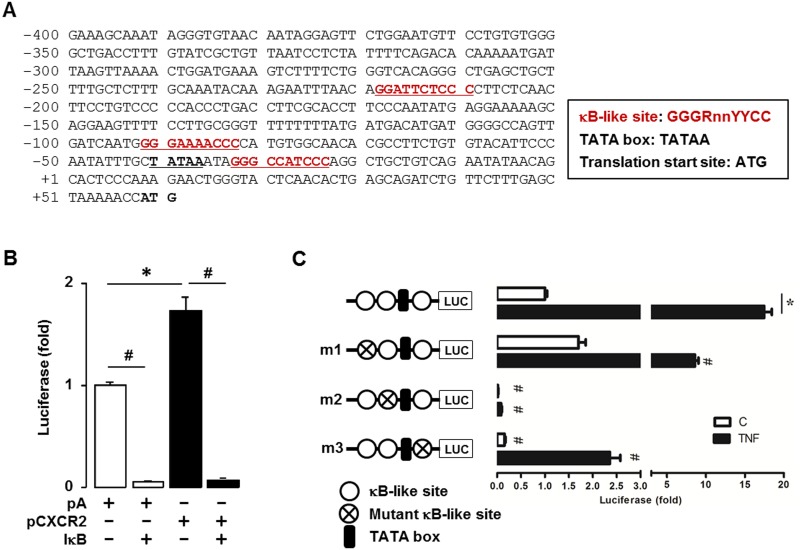
CCL20 promoter activity is tightly regulated by NF-κB signaling. (A) DNA sequence of human CCL20 promoter. (B) Increased effect of CXCR2 and blockage of IκB on luciferase activity of CCL20 promoter in parental SKOV-3 cells. Results were normalized to the protein level and expressed as a fold increase compared to empty vector (pA) controls. (C) Confirmation of CCL20 promoter activity and its mutants activity in response to TNF (10 ng/ml) in parental SKOV-3 cells. Site-directed mutants were generated from the CCL20-376/20LUC using primers with mutant κB-like sites (termed m1, m2 and m3 indicates mutation site): -219/-210 mutant κB-like site (CCL20LUCm1), -92/-83 mutant κB-like site (CCL20LUCm2) and -33/-24 mutant κB-like site (CCL20LUCm3). After transfection with CCL20 luciferase vectors overnight, a luciferase assay was performed at post-treatment of TNF (10 ng/ml) for 6 h. Results were normalized to the protein level and expressed as a fold increase compared to non-treated control (C). Cross circles indicate κB site mutants. pA = empty vector transfection; pCXCR2 = CXCR2 vector transfection. *,^#^ indicate significant (p≤0.05) increase and decrease, respectively, when a Student’s-*t* test was analyzed.

Although CXCR4 mRNA levels were increased in SKCXCR2-bearing tumors, the overall increased levels were still low (average cycle threshold >30) (Fig 3B). So as further studies, we focused on CCL20 of which mRNA levels were high (average cycle threshold <30) (Fig 3B). Based on higher NF-κB activation and CCL20 expression in the omental tumor tissues from SKCXCR2-bearing mice (Figs 2 and 3), we investigated if NF-κB signaling is critical in regulating CCL20 using parental SKOV-3 cells. The DNA sequence of CCL20 promoter was found to contain three κB-like sites as follows: distal (-219/-210), proximal (-92/-83) and post-TATA box (-33/-24) regions (Fig 4A). The CXCR2 vector-transfected cells increased the CCL20 promoter activity compared to cells transfected with empty vector (pA) (Fig 4B). On the other hand, IκB expression vector blocked CCL20 promoter activities at basal and CXCR2-induced levels (Fig 4B). Based on CCL20 (-376/+20) promoter as generated previously [6], we further mutated each κB-like site and identified which κB-like site is critical in regulating CCL20. TNF, a NF-κB activator, induced fully CCL20 promoter activity (Fig 4C). The mutation in each κB-like site abrogated the response of the promoter to TNF. The mutation of κB-like site at -219/-210 region reduced TNF-induced CCL20 promoter activity without decreasing the basal activity. The mutation of κB-like site at -33/-24 region decreased both basal and TNF-induced CCL20 promoter activity. In particular, the proximal region from -92 to -83 appeared to be the most critical site in regulating CCL20 even by eliminating TNF-induced CCL20 promoter activity, compared to other two κB-like sites (Fig 4C).

### Chemokine network of the omental tumor tissues exert a different pattern compared to that of parental cancer cells

**Fig 5:**
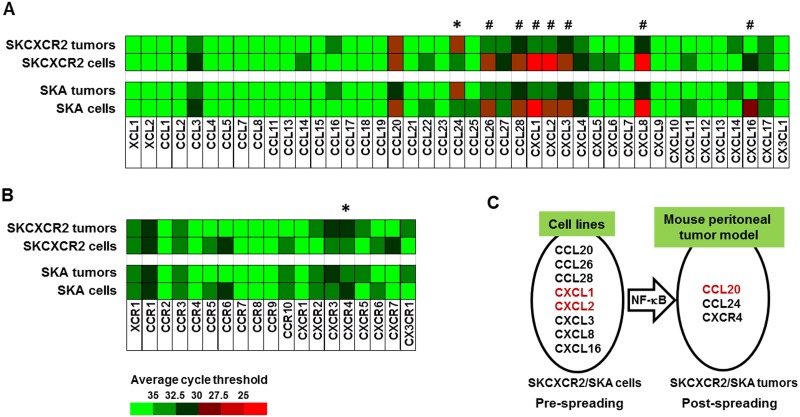
Shift of chemokine network in the tumor tissues of the omentum after implanted with parental cancer cells. (A) Chemokine and (B) chemokine receptor network in the tumor tissues of the omentum implanted vs. SKA and SKCXCR2 cell line. Expression levels of chemokine network are the means of 4 tumor tissues and cell lines in duplicate, respectively. *,^#^ indicate increased and decreased chemokines, respectively. (C) Proposed shift of dominant chemokines between pre-spreading and post-spreading of SKCXCR2 cells in the peritoneal cavity. Red letters indicate dominant chemokines in SKCXCR2 cells compared to SKA cells.

SKCXCR2 cells activated NF-κB at a greater degree compared to SKA cells, resulting in augmented proinflammatory chemokines CXCL1/2 as a target chemokine of NF-κB activation [18]. Because the omental tumor tissues expressed high mRNA levels of CCL20 instead of CXCL1/2, we compared chemokine network in the omental tumor tissues with that in the parental cells (SKA and SKCXCR2 cell line). Chemokines in the omental tumor tissues revealed some changes as follows: 1) the decreased levels in CCL26, CCL28, CXCL1-3, CXCL8 and CXCL16; 2) the increased level in CCL24; and 3) the conserved level in CCL20 compared to the parental cells (Fig 5A). On the other hand, all chemokine receptors in the omental tumor tissues and the parental cells were still in low levels although the increased level of CXCR4 mRNA was observed in SKCXCR2 omental tumor tissues (Fig 5B). The shift of dominant chemokines from CXCL1/2 to CCL20 is likely to occur during the peritoneal spreading of SKCXCR2 cells (Fig 5C).

### Different chemokine networks between human ovarian cancer cells and tumor tissues are similar to those in the mouse peritoneal spreading model for ovarian cancer

**Fig 6:**
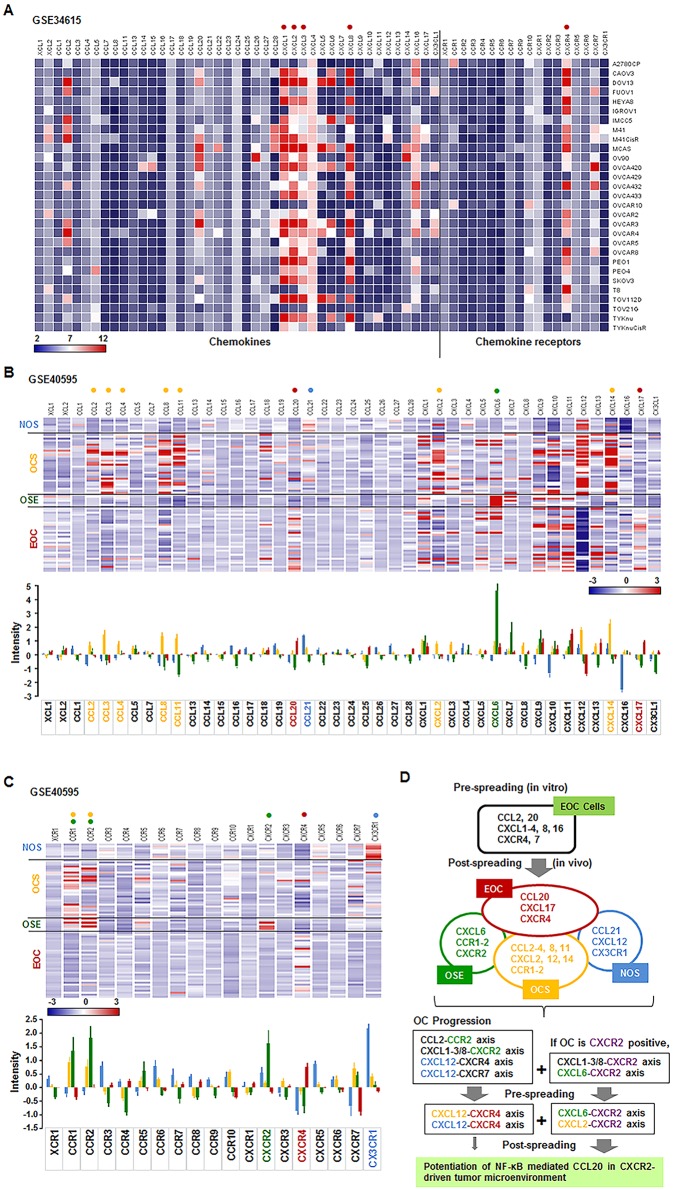
Profiles of chemokine network in human ovarian cancer cell lines and tumor tissues of the cancer associated fibroblasts. (A) Expression profiling array of chemokine ligand and receptor of 29 human ovarian cancer cell lines from datasets deposited in the NCBI Gene Expression Omnibus (GEO) database (GSE34615). Red dot indicates dominant chemokines in most of the cell lines used. (B) Chemokine and (C) chemokine receptor network in normal ovarian stroma (NOS), ovarian cancer stroma (OCS), ovarian surface epithelium (OSE) and epithelial ovarian cancer (EOC) from datasets deposited in the NCBI GEO database (GSE34615). The intensity of chemokine network was also analyzed. All values are presented as mean. Blue (NOS), yellow (OCS), green (OSE) and red (EOC) dots and letters in chemokine signature indicate dominant chemokine signatures (p≤0.05), respectively, as analyzed by ANOVA and Tukey’s pairwise comparisons. (D) Proposed shift of dominant chemokines between pre-spreading (*in vitro*) and post-spreading *(in vivo*) in ovarian cancer.

In line with the mouse peritoneal spreading model, we compared chemokine network between human ovarian cancer cells and tumor tissues based on GEO datasets. Chemokine signature of a panel of 29 human ovarian cancer cell lines showed dominant expression of CXCL1-3, 8 and CXCR4 (Fig 6A). Continually, we analyzed the chemokine network of normal ovarian stroma (NOS), ovarian cancer stroma (OCS), ovarian surface epithelium (OSE) and epithelial ovarian cancer (EOC) with high grade serous ovarian cancer tissues. The dominant chemokine signature in each compartment appears as follows: CCL21, CXCL12 and CX3CR1 for NOS; CXCL6, CCR1-2, CXCR2 for OSE; CCL2-4, 8, 11, CXCL2, 12, 14 and CCR1-2 for OCS; and CCL20, CXCL17 and CXCR4 for EOC (Fig 6B and 6C). Concurrently, the analyzed results showed that there is shift of dominant chemokines from CXCL1-3 and 8 (CXCR2 ligands) to CCL20 between pre-spreading (*in vitro*) and post-spreading (*in vivo*) in ovarian cancer (Fig 6D), as demonstrated in our mouse peritoneal spreading model. Probably, CXCR2 positive ovarian cancer cells could potentiate the NF-κB mediated CCL20 in proinflammatory tumor microenvironment via interaction between ovarian cancer and cancer-associated stromal cells compared to CXCR2 negative cells (Fig 6D).

## Discussion

Based on the mouse peritoneal xenograft model and human GEO datasets, the present study provides evidence that NF-κB mediated CCL20 is a dominant chemokine in the progression of ovarian cancer and is further potentiated even in CXCR2-driven ovarian progression which utilizes CXCL1-3 and 5–8 as CXCR2 ligands. Unexpected results enhancing mRNA levels of CCL20 rather than CXCL1/2 in CXCR2-driven ovarian progression indicate the diversity and complex of chemokine network between cell culture system with homogenous interaction and tumor progression via heterogeneous interaction with other cell types. The mouse peritoneal xenograft model has provided a valuable system for pre-clinical trials by reflecting clinical symptoms described in ovarian cancer progression such as peritoneal tumor dissemination and massive ascites [26]. Our peritoneal xenograft model revealed a greater tumor burden in SKCXCR2-bearing mice compared to SKA-bearing mice, based on short survival time, wide peritoneal tumor spreading and increased tumor weight (Fig 1). Consistent with our results, silencing CXCR2 inhibited subcutaneous tumor formation in nude mice injected with SKOV-3 cells [11]. Stable transfection of CXCR2 into SKOV-3 cells accelerated cell proliferation, migration and invasion in cell culture system [18]. Furthermore, overexpression of CXCR2 had poor overall and disease-free survival of patients with high-grade serous ovarian carcinoma [11]. These facts suggest that CXCR2 can prompt clinical symptoms such as peritoneal tumor dissemination and massive ascites followed by a high mortality.

Particularly, the NF-κB mediated signaling, a well-established dominant transduction pathway for the development and progression of a variety of tumors, was involved in CXCR2-driven ovarian cancer progression (Fig 2). On the other hand, unexpectedly, there was no difference in EGFR-activated signaling pathway and its downstream components between SKA- and SKCXCR2-derived tumor tissues (Fig 2). Several *in vitro* cell culture experiments showed evidence of CXCR2-mediated EGFR transactivation. Silencing CXCR2 decreased Erk activation in SKOV-3 cells [11]. CXCL1 functions through CXCR2 to transactivate the EGFR by proteolytic cleavage of HB-EGF, leading to activation of MAPK in SKOV-3 cells [27]. Overexpression of CXCR2 increased EGFR-transactivated Akt and Erk in ovarian cancer cells such as SKOV-3 and OVCAR-3 [18]. The different expression profiles of chemokines between parental and implanted cancer cells indicates the alteration of dominant signaling pathways, probably via interaction with other cells in the tumor microenvironment during implanting and spreading process of cancer cells. The CXCR2-mediated pathway is likely to involve NF-κB signaling, supported by the report that silencing CXCR2 decreased NF-κB signaling in SKOV-3 cells [11]. Also, our recent study demonstrated that CXCR2 potentiated NF-κB activation through EGFR-transactivated Akt [18]. In spite of no change in EGFR activation after cancer spreading of SKA and SKCXCR2 cells, the NF-κB signaling pathway appears to conserve CXCR2-driven ovarian cancer progression throughout the whole process including initiation, promotion and progression phases.

NF-κB exerts various tumor-promoting functions through activation of distinct target genes including proinflammatory chemokines [28]. Our previous study demonstrated that proinflammatory chemokines such as CCL20, CXCL1-3 and CXCL8 are elicited in ovarian cancer cells via NF-κB- and EGFR-activated signaling pathways [6]. Furthermore, as described in detail previously [18], CXCR2 positive ovarian cancer cells enhanced more the promoter activity and mRNA levels of CXCL1 and 2 by NF-κB potentiation through EGFR-activated Akt compared to CXCR2 negative cells. These facts support the expectation that NF-κB is mainly involved in the progression of CXCR2-driven ovarian cancer, leading to augmentation of CXCR2 ligands such as CXCL1-3 and 5–8 followed by proinflammatory tumor microenvironment via CXCR2 axis. Therefore, to clarify CXCR2 axis in the progression of CXCR2-driven ovarian cancer, we performed the comprehensive chemokine network analysis in the ovarian tumor tissues using PCR array. Different from cell culture system where CXCL1 and 2 were enhanced [18], CCL20 was specifically increased in SKCXCR2-derived tumor tissues compared to SKA-derived tumor tissues (Fig 3). Unexpectedly, CXCR2 ligands such as CXCL1-3 and 8 were decreased in the progression of CXCR2-driven ovarian cancer compared to relatively constant CCL20 levels (Fig 5). Although chemokine network is complex, it’s evident that a single chemokine can impact the direction of the spreading of a cancer cell [29–31]. Although CXCR4 mRNA was increased in SKCXCR2-derived tumor tissues, its enhanced levels were still low (Fig 3). So we prioritized CCL20 for further discussion. CCL20 was highly expressed in several cancer types such as colorectal malignancies [32, 33], metastatic triple-negative breast cancer [34], non-small-cell-lung-cancer [35], hepatic malignancies [36] and pancreatic carcinoma [37]. Furthermore, the CCL20-CCR6 axis recruited circulating regulatory T cells into tumor microenvironment, resulting in tumor progression and poor prognosis in patients with hepatocellular carcinoma [38]. Overexpression of CCL20 in prostate cancer cells promoted tumor growth and invasiveness [39]. In addition, CCL20 increased the migration and invasiveness of MDA-MB-231 breast cells, but did not affect the cell proliferation [40]. These facts support the observation that a high expression of CCL20 in SKCXCR2-derived tumor tissues can be involved in the progression of ovarian cancer. Particularly, our previous study demonstrates that SKOV-3 cells had a synergistic response in CCL20 and CXCL8 levels when exposed to EGF plus TNF [6]. Therefore, high levels of TNF [2–4] and frequent overexpression of EGFR observed in ovarian cancer [41, 42] could provide the fundamental basis for the higher expression of CCL20 in SKCXCR2-derived tumor tissues (Fig 3).

Chemokine ligand at the metastatic site can initiate pro-tumor inflammatory network, delivering anti-apoptotic and proliferative signals that encourages tumor cells to grow [43]. It is clear that chemokines are implicated in aggressive metastatic tumor progression and that a better understanding of their regulation could lead to new therapeutic targets for cancer. The results from our present study suggest that NF-κB is an important signaling for the induction of CCL20 as shown higher activation of NF-κB and expression of CCL20 in SKCXCR2-derived tumor tissues (Figs 2 and 3). NF-κB like sites have been found in the promoter regions of cytokines and chemokines [44] and the activation of NF-κB has been shown to be important for their induction [45]. Therefore, we first asked whether NF-κB plays critical role in CXCR2-mediated CCL20 regulation to elucidate the signaling pathway involved in the induction of CCL20 during ovarian cancer progression. We identified three potential κB-like sites in CCL20 promoter region and found that κB-like site located between -92 and -83 bp upstream from the transcriptional site is most critical in regulating CCL20 (Fig 4C). Other cell types also support the importance of NF-κB site in regulating CCL20. NF-κB site is essential to CCL20 promoter activity in intestinal cell lines Caco-2 and T84 [46]. TNF-induced CCL20 is conferred by a region between -111 and -77, which contains a κB-like site in G-361 human melanoma cells [47].

In contrast to the increase of CCL20 in SKCXCR2-derived tumor tissues *in vivo*, SKCXCR2 cells *in vitro* had specific increase in CXCL1/2 through NF-κB signaling compared to SKA cells [18]. It indicates the shift of dominant chemokine network in the progression of ovarian cancer throughout initial, implanting and spreading stages. During the peritoneal spreading of SKCXCR2 cells, dominant chemokines are likely shifted from CXCL1/2 to CCL20. In addition, to confirm our results from the mouse peritoneal xenograft model, we analyzed the data of the chemokine signatures in human ovarian cancer cell lines, and ovarian tissues (surface epithelium and stromal cells) obtained from healthy and ovarian cancer patients based on GEO datasets. The human ovarian cancer cell lines (pre-spreading *in vitro*) showed dominant expression levels of CXCL1-3, 8 and CXCR4, while EOC (post-spreading *in vivo*) had higher levels of CCL20, CXCL17 and CXCR4. These results support our findings that there is a shift of dominant chemokines from CXCL1/2 to CCL20 between pre- and post-spreading of ovarian cancer. Interestingly, tumor tissues derived from SKA-/SKCXCR2 induced greater CCL24, compared to cultured cells from SKA/SKCXCR2 (Fig 5). The elevated level of CCL24 was found in primary colorectal cancer biopsies [48]. However, increasing evidence indicates that CCL24 is important for recruitment of eosinophils in mice challenged with ovalbumin [49] and in human with intradermal injection of CCL24 [50]. Regulation of CCL24 is likely to involve specific transcription factors such as Smad3 and GATA-1. CCL24 was increased in the skin of Smad3-/- mice [51] and in the GATA-1-overexpressed human leukemic HT93 cell line [52]. Although TNF induced CCL24 protein in epithelial cells of nasal polyps [53], it had no effect in SKOV-3 cells [5, 7]. Therefore, it is still unclear whether NF-κB signaling is directly involved in regulating CCL24, requiring further studies.

Based on the results from the mouse peritoneal xenograft model and human GEO datasets, we summarized the characteristics of the chemokine network between cell culture system and tumor tissues, and described the development of expected chemokine network for cell-cell communication in the tumor microenvironment during the progression of ovarian cancer (Fig 6D). Ovarian cancer cell culture system or pre-spreading local growth lacking extensive interaction with other cell types appears to express high levels of CCL2, 20, CXCL1-3, 8 and 16, and CXCR4 and 7. These chemokine signatures in EOC cells appear to shift during the peritoneal spreading, leading to dominant expression of CCL20 and CXCR4 through cell-cell communication in the tumor microenvironment as follows: CCL2-CCR2 and CXCL1-3/8-CXCR2 axes via interaction with OSE, and CXCL12-CXCR4 and CXCL12-CXCR7 axes via interaction with NOS. Post-spreading EOC tumors can develop CXCL12-CXCR4 axis via interaction with NOS and OCS. In addition to these axes, CXCR2 positive EOC will involve the following chemokine axes: CXCL1-3/8-CXCR2 axis via autocrine manner and CXCL6-CXCR2 axis via interaction with OSE in pre-spreading status of EOC, and CXCL6-CXCR2 axis via interaction with NOS and CXCL2-CXCR2 axis via interaction with OCS in post-spreading status of EOC. Finally, EOC expressing CCL20 and CXCR4 could easily chemoattract CCR6 (CCL20 receptor) expressing cells and metastasize to CXCL12 (CXCR4 ligands) expressing area.

In conclusion, CXCR2-driven ovarian cancer progression in the peritoneal cavity involves NF-κB mediated CCL20 as a main chemokine network, which would lead to new approaches of ovarian cancer progression and treatment.

## Supporting Information

S1 Fig Changes in body weight of mice after the peritoneal inoculation of SKA and SKCXCR2 cells. Body weight of the mice increased because of tumor growth and development of ascites. We observed 3 dead (SKA_3,4,8) in 8 SKA-bearing mice and 4 dead (SKCXCR2_2,3,4,5) in 8 SKCXCR2-bearing mice.
(TIF)
